# Current Advances in Chitosan Nanoparticles Based Drug Delivery and Targeting

**DOI:** 10.15171/apb.2019.023

**Published:** 2019-06-01

**Authors:** Unnati Garg, Swati Chauhan, Upendra Nagaich, Neha Jain

**Affiliations:** Amity Institute of Pharmacy, Amity University, Sector-125, Noida, Uttar Pradesh-201303.

**Keywords:** Chitosan, Nanoparticles, Biodegradable, Applications, Drug Delivery

## Abstract

Nanoparticles (NPs) have been found to be potential targeted and controlled release drug delivery systems. Various drugs can be loaded in the NPs to achieve targeted delivery. Chitosan NPs being biodegradable, biocompatible, less toxic and easy to prepare, are an effective and potential tool for drug delivery. Chitosan is natural biopolymer which can be easily functionalized to obtain the desired targeted results and is also approved by GRAS (Generally Recognized as Safe by the United States Food and Drug Administration [US FDA]). Various methods for preparation of chitosan NPs include, ionic cross-linking, covalent cross-linking, reverse micellar method, precipitation and emulsion-droplet coalescence method. Chitosan NPs are found to have plethora of applications in drug delivery diagnosis and other biological applications. The key applications include ocular drug delivery, per-oral delivery, pulmonary drug delivery, nasal drug delivery, mucosal drug delivery, gene delivery, buccal drug delivery, vaccine delivery, vaginal drug delivery and cancer therapy. The present review describes the formation of chitosan, synthesis of chitosan NPs and their various applications in drug delivery.

## Introduction

**Figure 1:**
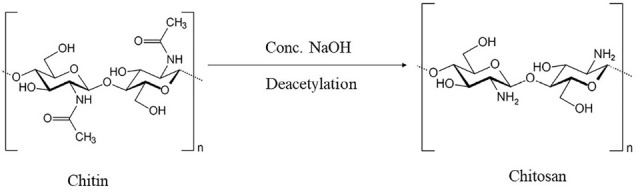
Deacetylation of chitin to chitosan.

Nanoparticles (NPs) can be defined as submicron colloidal drug carrier systems which are composed of natural or artificial polymers ranging in size between 10 and 100 nm. NPs offer numerous advantages as compared to microparticles such as sustained and controlled drug release, site-specific targeting and high surface to volume ratio. These properties help in reducing the required drug dose and frequency of administration which improves patient compliance.^1^ The Chitosan NPs are biodegradable, more stable, simple, less toxic, biocompatible and easy to prepare. These are made of a fully biodegradable and biocompatible natural polymer chitosan and also approved by GRAS (Generally Recognized as Safe by the United States Food and Drug Administration [US FDA]).^2^ The chitosan NPs are soluble in aqueous acidic solution thereby avoiding the use of hazardous organic solvents. Also, they have the ability to control the release of the active agents.^3^ Chitosan is a mucopolysaccharide and is closely related to cellulose It is a derivative of chitin and is produced by the deacetylation of chitin. The structure of chitin deacetylation is shown in Figure 1. It is derived from crustacean shells as well as from cell walls of fungi. Chitosan has a unique feature i.e. the presence of the primary amine at the C-2 position of the glucosamine residues. The presence of this much high amines provides important functional properties to chitosan.^1,4^ The degree of deacetylation and the molecular weight of chitosan affect its physico-mechanical properties. The degree of deacetylation affects its hydrophobicity, solubility and toxicity. The chitosan having higher degree of deacetylation shows toxicity according to their molecular weights i.e. are less toxic for low molecular weight and high toxic for high molecular weights. However, the chitosan having lower degree of deacetylation acts as an absorption enhancer at both high and low molecular weights. The molecular weight of chitosan affects its solubility and degradation. Chitosan with higher molecular weights show less solubility and lower degradation rate than the chitosan with lower molecular weight.^5^ Chitosan is composed of different elements which include Carbon (44.11%), Hydrogen (6.84%) and Nitrogen (7.97%). The viscosity average molecular weight of chitosan is ~5.3X10^5^ Daltons.^6^ The chemical functional groups present in chitosan can be modified to achieve a specific goal, thereby expanding its range of potential applications. NPs which are prepared from chitosan and its derivatives generally possess a positively charged surface. Chitosan also possesses some limitations along with its so many advantages as highlighted in Table 1. These include, low solubility in neutral and alkaline pH and the method of preparation has to be changed according to the drug to be delivered.^7^ Chitosan NPs are prepared by different methods like ionic cross-linking method, covalent cross-linking method, reverse micellar method, precipitation and emulsion-droplet coalescence method.^4^ Chitosan NPs have various potential application viz parenteral drug delivery, ocular drug delivery, mucosal drug delivery, per-oral drug delivery, gene delivery, vaccine delivery, cancer therapy, pulmonary drug delivery, intranasal drug delivery, etc. A plethora of clinical studies are going on to investigate the biocompatibility, safety and efficacy of chitosan NPs as dosage form.

**Table 1:** List of advantages and drawbacks of chitosan nanoparticulate dosage form

**Advantages**	**Disadvantages**
Toxicity is less	Mechanical resistance is less
Enhanced Biocompatibility	Difficulty in controlling pore size
Possess mucoadhesive character	May contract
Possess stability	Electrospinning is difficult for pure chitosan
Drug targeting is site-specific	Preparation by crosslinking can affect intrinsic properties of chitosan
Therapeutic index of the drug is increased	Low solubility in neutral and alkaline pH
Frequent, expensive and unpleasant dosing is prevented	Method of preparation must be changed according to the drug to be delivered

Improvement of physicochemical properties of chitosan can also enhance the utility of chitosan NPs. Chitosan NPs have different properties which can further be enhanced for more efficient drug-delivery. For improving the intestinal solubility of chitosan, N-trimethyl chitosan chloride has been produced. To increase the mucoadhesiveness of chitosan, NPs with thiolated chitosan were formulated. Chitosan acts by opening the tight junctions of the membrane, so quaternization of chitosan enhances this effect as in case of insulin permeation across Caco-2 cells. PH sensitivity can be achieved by grafting carboxylated chitosan with poly (methyl methacrylate). For specific applications, physical modifications can be done in chitosan by blending i.e. physically mixing two or more polymers. For example, blending of chitosan with poly vinyl alcohol improves the mechanical and barrier properties of chitosan.^7^

## Preparation of chitosan nanoparticles

NPs are particulate dispersions or solid particles with a size range of 1-1000 nm. Methods such as ionic cross linking; covalent cross linking, reverse micellar method can be used for the preparation of chitosan NPs.

## Ionic cross-linking method

**Figure 2:**
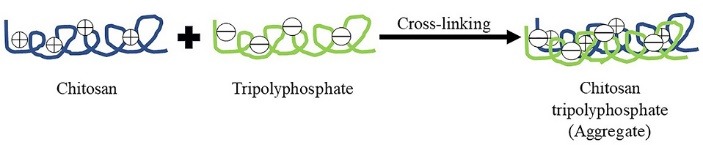
Diagrammatic representation of chitosan nanoparticles preparation by ionic cross-linking technique.

In this method ionic cross-linking is achieved by aggregation of chitosan or its derivatives with oppositely charged macromolecules or in the presence of ionic cross-linking agent. Tripolyphosphate is the most commonly used cross-linking agent. There is a formation of gels due to ionic linkage, therefore this method is also known as ionic-gelation method^8^ as outlined in Figure 2. Chitosan-tripolyphosphate ionotropic gelation is used for the preparation of estradiol (E2)-loaded chitosan NPs which have zeta potential equal to +25.4 mV and an average size equal to 269.3±31.6 nm.^5^

## Covalent cross-linking method

In this method there is formation of covalent bonds between chitosan or its derivatives and the functional cross-linking agent. Commonly used agents include polyethylene glycol, glutaraldehyde or monofunctional agents.^9^

## Reverse micellar method

**Figure 3:**
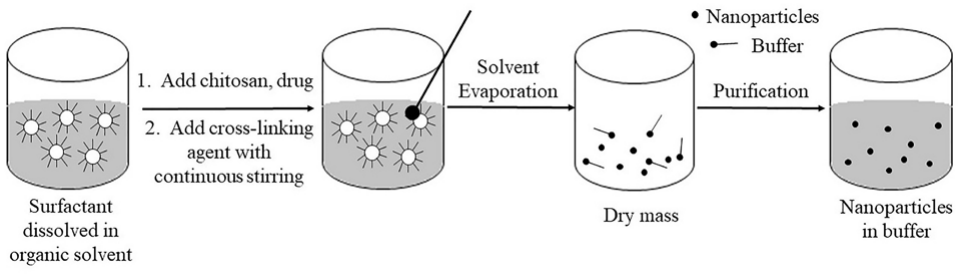
Diagrammatic representation of chitosan nanoparticles preparation by reverse micellar method.

In this method, an aqueous solution of chitosan is added to an organic solvent containing a surfactant. Agitation is done simultaneously. Water is added to maintain the mixture in an optically transparent microemulsion phase. The amount of water is increased to obtain NPs of larger size as shown in Figure 3. Chitosan NPs prepared by this method have been used for encapsulation of doxorubicin-dextran conjugate.^4^

## Precipitation/coacervation

**Figure 4:**
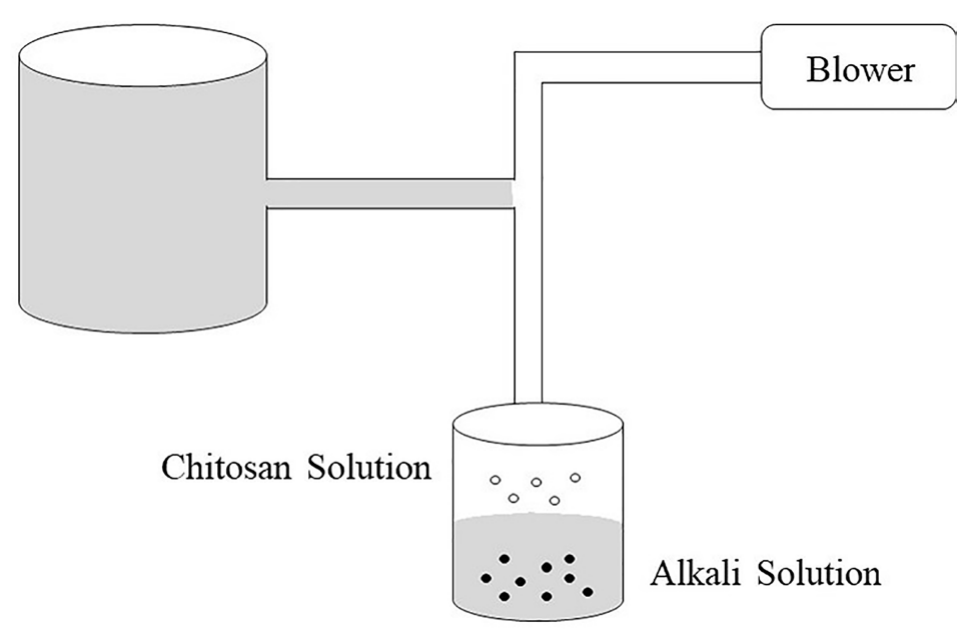
Illustration representing the preparation of chitosan nanoparticles by coacervation/precipitation.

This method is based on the utilization of physicochemical properties of chitosan i.e. its insolubility in alkaline pH medium and thereby forming a precipitate. In this method, the chitosan solution is blown into an alkali solution using a compressed air nozzle forming coacervate droplets. The particles are then separated and purified by filtration or centrifugation followed by successive washing with hot and cold water as depicted in Figure 4. This technique is used to prepare chitosan-DNA NPs.^4^

## Emulsion-droplet coalescence method

**Figure 5:**
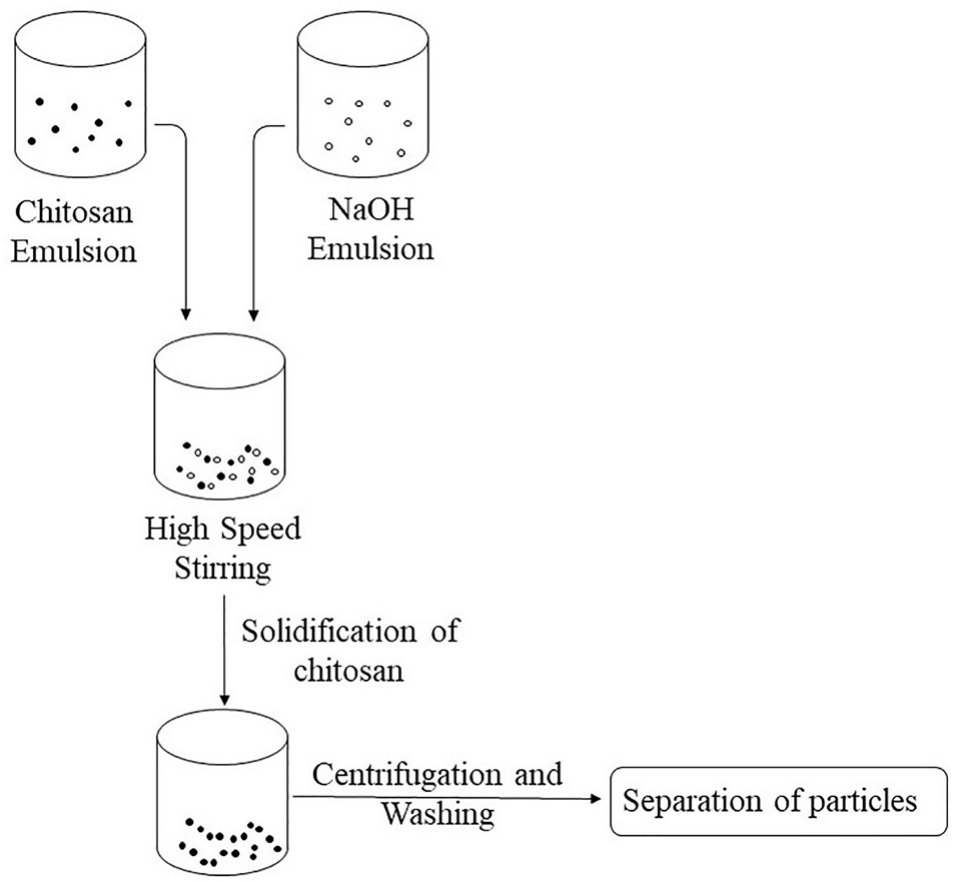
Diagrammatic representation of preparation of chitosan nanoparticles by emulsion droplet coalescence method.

This method was developed by Tokumitsu et al.^10^ It is based on the principle of emulsion cross-linking and precipitation. In this method, two emulsions are prepared, one containing aqueous solution of chitosan and drug in liquid paraffin oil and the other containing aqueous chitosan solution of NaOH in liquid paraffin oil. Both the emulsions are mixed under high speed stirring and there will be formation of droplets which would collide and coalesce, thereby precipitating chitosan droplets to give small size particles as illustrated in Figure 5. This method was used to prepare gadopentetic acid-loaded chitosan NPs.^4^

## Applications of chitosan nanoparticles

Chitosan NPs have numerous applications in pharmaceutical, biological and medical discipline. Some are enlisted as ocular drug delivery, per-oral delivery, pulmonary drug delivery, nasal drug delivery, mucosal drug delivery, gene delivery, buccal drug delivery, vaccine delivery, vaginal drug delivery, cancer therapy.

## Ocular drug delivery

Chitosan possesses the favorable characteristics such as nontoxicity, biodegradability and biocompatibility, which make it a suitable choice for ocular drug delivery systems. Also, it is mucoadhesive in nature, which increase the ocular surface duration of drugs.^11^ Chitosan also possesses in situ gelling properties due to which, when it is applied on the ocular surface in liquid form, it gets transformed into gel later. This leads to the improvement of the ocular residence time and therapeutic efficacy of the drug.^11,12^ Ping Zhang et al prepared sulfobutylether-β-cyclodextrin/chitosan NPs (Nag-CD/CS-NPs) containing naringenin and studied their topical ophthalmic delivery efficacy. They prepared NPs by first complexing naringenin with sulfobutylether-β-cyclodextrin (SBE-β-CD) and then performing ionic gelation of chitosan with SBE-β-CD. The average size of the resulting NPs was found to be 446.4 ± 112.8 nm and zeta potential was +22.5 ± 4.91 mV and was spherical in shape. They investigated there in vitro and in vivo properties. They carried out the in vivo study in rabbit’s eye and found out that the NPs were nonirritating and were able to prolong the residence time of naringenin which in turn increased its bioavailability in the aqueous humor. It was concluded that the chitosan NPs offer a potential alternative for the ocular administration of poorly soluble drugs.^13^ Santhi et al developed fluconazole loaded chitosan NPs using spontaneous emulsification and cross-linking method. They used cup-plate method to compare the anti-fungal properties of these NPs with the normal eye drops. The average size of these particles was found to be 152.85±13.7 nm. The drug loading capacity of all drug loaded NPs was found to be optimum (≤50%). After all their studies, they concluded that the formulated chitosan NPs of fluconazole were a suitable carrier for the ocular delivery of fluconazole in terms of optimum drug loading, antifungal activity and sustained release characters.^14^

## Per-oral drug delivery

NPs have various advantages such as small particle size, large surface area, and a potentially modifiable surface which prevent the enzymatic degradation of the drugs in the GIT; therefore these are used as oral delivery systems for polynucleotides, proteins and macromolecules. Also, they increase the GIT stability of the acid labile drugs.^15^ Chitosan has an absorption promoting effect due to its mucoadhesion properties and transient opening of the tight junctions of the mucosal cell membrane. Also, the interaction between chitosan (positively charged) and mucin (negatively charged) leads to an increase in contact time between the drug and the absorptive surface. Also, chitosan increases the half time of clearance due to its mucoadhesion.^16^ The ability of chitosan to increase the membrane permeability depends upon its degree of deacetylation and molecular weight. As the degree of deacetylation increases, the charge density of chitosan also increases which in turn improves the drug transportation, which means that there will be increased epithelial permeability. High molecular weight of chitosan also increases epithelial permeability.^17^ S. Barbieri et al developed a chitosan and phospholipid based nanoformulation loaded with tamoxifen (anti-cancer drug). When this formulation was administered orally, there was a 1.5 to 90 times increase in permeation in absence or in presence of pancreatin or lipase, respectively.^18^ Feng et al also prepared NPs of doxorubicin hydrochloride with chitosan and carboxymethyl chitosan which are used for treatment of cancer. These nanostructures were found to increase the intestinal absorption of doxorubicin hydrochloride throughout the small intestine.^19^

## Pulmonary drug delivery

Drug delivery to the lungs has various advantages such as rapid and sustained drug delivery, high efficacy, no first pass metabolism and both local and systemic effects can be achieved. The factors which contribute to the enhanced drug delivery via lungs are large surface area of lungs, thin absorption barrier and high vascularity.^20^ Islam and Ferro in their review claimed that drug delivery to the lungs can also be achieved by chitosan-based NPs. The positive charge of the chitosan provides it mucoadhesive properties, which increases potential for drug absorption and this positive charge also open the tight junctions which lead to an increased uptake. Chitosan also possesses antibacterial properties which provide an additional benefit of fighting the pulmonary bacterial infections.^21^ The effects of various drugs have been improved with the help of chitosan. Using chitosan as a polymer a nanoparticle dry powder inhalation of rifampicin, an anti-tubercular drug, was formulated. This formulation has shown sustained drug release until 24 hour and no toxic effects were shown to both the cell and organ.^22^ Jafarinejad et al developed chitosan NPs for the pulmonary delivery of itraconazole (anti-fungal drug) as a dry powder formulation due to its low solubility when administered orally. They formulated the drug with chitosan NPs, lactose, mannitol and leucine, which improved its aerosolization properties. This resulted in an increased pulmonary deposition of intraconazole.^23^ Petkar et al developed octanoyl chitosan NPs loaded with rifampicin for pulmonary delivery. This development improved sustained release and stability of rifampicin.^24^ Debnath et al administered prothionamide (antitubercular drug) in the form of nanoparticle via pulmonary route and these NPs were coated with chitosan. This modification improved the residence time of the drug in the lungs.^25^

## Nasal drug delivery

**Figure 6:**
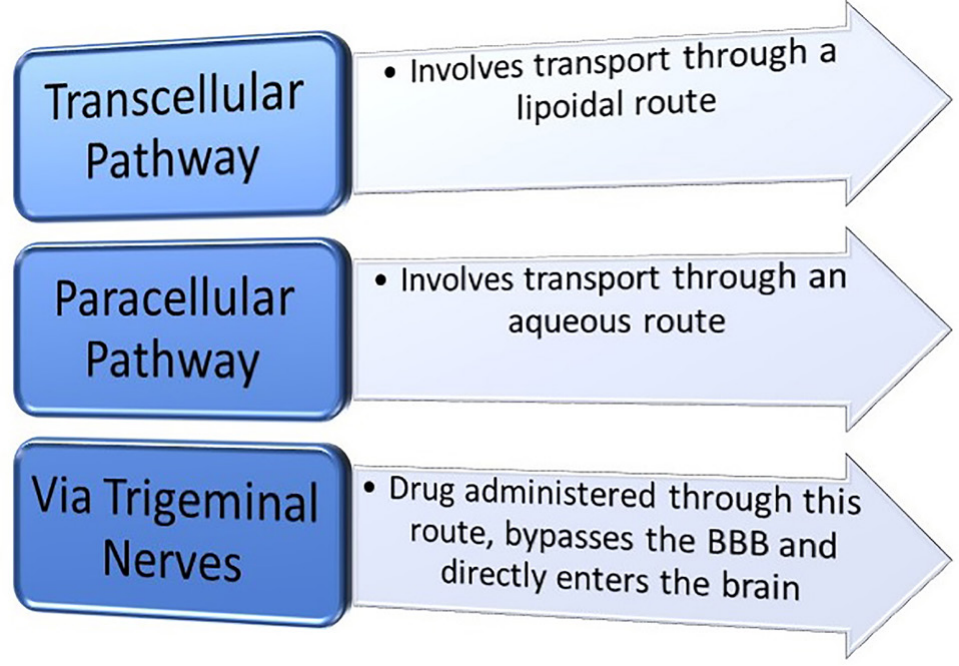
Different ways of nasal absorption.

Nasal delivery is a non-invasive technique through which, the delivered drugs reach the brain, the respiratory system and/or the systemic circulation. Chitosan increases the permeability of the hydrophilic drugs, nucleic acids, proteins and peptides across the nasal epithelium as they pose difficulty due to their low permeability. Certain characteristics such as weight, lipophilicity and charge of the drug affect its nasal absorption. The drugs which are unable to cross the nasal membrane undergo mucociliary clearance. A mucoadhesive system can help to overcome this problem and chitosan possesses mucoadhesion properties along with low toxicity, biodegradability and biocompatibility, owing to its application in nasal delivery.^26^ Nasal absorption can take place by three ways i.e. transcellular pathway, paracellular pathway and via trigeminal nerves as depicted in Figure 6.^27^ Liu et al proposed the use of carboxymethyl chitosan NPs as a carrier to deliver carbamazepine (anti-epileptic drug) intra-nasally which will bypass the blood brain barrier thereby enhancing the brain drug concentration and therapeutic efficacy. They developed NPs which were found to have small particle size (218.76 ± 2.41 nm) with high entrapment efficiency (80%). They performed in vivo and in vitro tests which showed increased drug bioavailability and enhanced brain targeting characteristics.^28^ Shahnaz et al developed leuprolide loaded chitosan and thiolated-chitosan NPs. These chitosan and thiolated-chitosan NPs increased the drug transport across the porcine nasal mucosa from 2 to 5 folds respectively than the leuprolide solution. The drug exposure increased by 6.9 fold with thiolated-chitosan NPs as measured by area under the plasma concentration vs time curve AUC.^29^

## Mucosal drug delivery

Chitosan and its derivatives enhance absorption of hydrophilic molecules such as protein and peptide drugs, thereby helping in improvement of mucosal delivery. Mucus is composed of highly hydrated glycoproteins (salts, lysozymes and mucins) which are responsible for its viscoelastic properties. Mucinhas sialic acid residues which have a pK_a_ equal to 2.6 which makes it negatively charged at physiological pH.^30,31^ Chitosan acts by opening the intercellular tight junctions, causing the paracellular transport of macromolecular drugs.^32^ The chitosan NPs are positively charged and bind to the cell membrane and decrease the transepithelial electrical resistance of cell monolayers and also increase the paracellular permeability. The chitosan solution increases the trans and para-cellular permeability depending upon the molecular weight and degree of deacetylation of chitosan.^32^ The mechanism of action used by chitosan involves the interaction of its positive charge with the tight junction proteins i.e. occludin and ZO-1, redistribution of F-actin and slight destabilization of plasma membrane. Also, it has been shown that the ability of chitosan to enhance the permeation is also influenced by the environment.^33,34^ Martirosyan et al utilized the mucoadhesive properties of chitosan for the delivery of siRNA across the mucosal surfaces following local administration.^35^ Prego et al reported that chitosan NPs increased the intestinal absorption of salmon calcitonin, due to their mucoadhesive character and intimate interaction with the intestinal barrier.^36^

## Gene delivery

In gene delivery process, the plasmid DNA is introduced in the target cells, which is then transcribed and the genetic information is finally translated into the corresponding protein. For this process to be completed, the gene delivery system has to overcome a number of hurdles. The various factors affecting transfection include, targeting the delivery system to the target cell, uptake and degradation in the endolysosomes, transport through the cell membrane and intracellular trafficking of plasmid DNA to the nucleus. Chitosan protects the DNA against nuclease degradation by forming a polyelectrolyte complex with the negatively charged DNA. This protection improves the transfection efficiency.^4^ Gene delivery can be done with the help of viruses as well as without them. Some investigations are compiled in Table 2.^37-39^ Although, viruses can transfer genes into cells efficiently^40,41^ but due to safety concerns after the death of Jesse Gelsinger in 1999 due to an adenoviral vector, non-viral gene vectors are preferred.^42,43^ Cationic polymers and lipids are considered to be promising gene delivery agents for large scale production and commercial use as non-viral vectors.^44-46^ Chitosan is a cationic polysaccharide which exists naturally and binds strongly to DNA.^47,48^ Li et al prepared quaternary chitosan by modification of quaternary ammonium group to improve the DNA carrying capacity and solubility of chitosan.^49^ Deng et al, for the treatment of triple negative breast cancer, prepared miR-34a (endogenous tumor suppressive molecule) and doxorubicin loaded hyaluronic acid-chitosan NPs. They used ionotropic gelation method for the preparation of these NPs. The conducted in vivo and in vitro studies and concluded that, hyaluronic acid-chitosan was a successful dual nanocarrier system to deliver both doxorubicin and miRNA-34a into triple negative breast cancer cells for improved chemotherapy efficacy.^37^ Rudzinski et al developed chitosan and poly ethylene glycol-grafted NPs loaded with anti-β-catenin siRNA for transfection into colon cancer cells. They performed western blot analysis for both the particles and found that these NPs were successfully entering the colon cancer cells and thus decreasing the level of protein that promotes tumor progression.^38^

**Table 2:** Investigations based on chitosan nanoparticles in gene delivery

**Modifications made in Chitosan NP**s	**Inference**	**Reference**
Quaternary chitosan by modification of quaternary ammonium group	Improved DNA carrying capacity and solubility of chitosan	37
miR-34a and doxorubicin loaded hyaluronic acid-chitosan NPs	Successful dual nanocarrier system to deliver both doxorubicin and miRNA-34a into triple negative breast cancer cells for improved chemotherapy efficacy	38
Chitosan and polyethylene glycol-grafted NPs loaded with anti-β-catenin siRNA	Successful in entering the colon cancer cells and thus decreasing the level of protein that promotes tumour progression	39

## Buccal drug delivery

Buccal drug delivery has become a preferred route because of its various advantages such as bypassing the first pass metabolism which increases the drug bioavailability and reduces the amount of drug required. Also, through this route the drugs of high molecular weight can be delivered which cannot be administered by the oral route^39^ and the drug is not exposed to the harsh gastro intestinal environment.^50^ Ideally, a buccal drug shows controlled drug release and a prolonged time of residence in the oral cavity.^51^ Mazzarino et al developed curcumin-loaded chitosan-coated polycaprolactone NPs contained in mucoadhesive films to increase the residence time of the drug in the oral cavity and to enhance the drug absorption through buccal mucosa. They performed atomic force microscopy and high‐resolution field‐emission gun scanning electron microscopy (FEG-SEM). From the results they concluded that, mucoadhesive films with NPs are a good approach for the buccal delivery of curcumin which is useful in the treatment of periodontal diseases.^52^

## Vaccine Delivery

Chitosan has been widely used for mucosal and systemic vaccine delivery and also in preparation of DNA mucosal vaccines. It is used in mucosal vaccine delivery as it promotes absorption which enhances mucosal immune response.^32,53,54^ Mainly, the vaccine administered through oral route requires the use of NPs. Nasal route vaccines are also there. In oral delivery vaccine the main target are Peyer’s patches. Due to the nanoparticle system, the vaccine is protected from enzymatic degradation while going to the mucosal tissue and taken up by the M-cells. On the other hand, the nasal administered vaccines remain in the nasal cavity for about 15 minutes only; have to be transported over a very small distance and to be protected from low pH and enzymatic degradation.^1^ Chitosan vaccines containing influenza, diphtheria and pertussis antigens for nasal delivery were prepared by Illum et al.^55^ In systemic vaccine delivery, chitosan acts as an adjuvant. Activation of macrophages occurs after the uptake of chitosan.^32,53,54^ Chitosan has been widely used for DNA mucosal vaccines. Illum et al. developed a chitosan-based DNA flu vaccine.^55^ Incorporation of plasmid pCMVArah2 encoding peanut allergen gene into chitosan NPs showed good antigen expression and good protection after oral administration in mice.^1,56,57^ Vaccine association with particulate systems such as NPs, enhances the antigen uptake by mucosal lymphoid tissue, which induces strong mucosal and systemic immune responses against the antigens.^58^ Pawar and Jaganathan prepared glycol chitosan NPs for the intranasal delivery of hepatitis B vaccine. They obtained NPs with high loading efficiency i.e. >95% and an average particle size of 200 nm. They performed in vivo and in vitro studies and the results concluded that these NPs have a good potential for the mucosal delivery of vaccines.^59^ Marasini et al developed four types of lipopeptide antigen-loaded polymeric NPs to determine which is the most suitable system for the delivery of lipopeptide-based vaccine against group A *Streptococcus*. They performed different tests and based on the results, they concluded that the lipopeptides loaded dextran/trimethyl chitosan NPs were efficient for the intranasal delivery of lipopeptide-based vaccines.^60^

## Vaginal drug delivery

The vaginal route is very helpful in offering both local and systemic effects^61,62^ and has various advantages such as bypassing the first pass metabolism and avoiding the acidic pH of the stomach. The vaginal mucosa is easily accessible and has a rich blood supply.^63,64^ Chitosan has properties such as facilitating mucoadhesion, capability to interact with the mucous layer and increasing the drug residence time at the mucosal surface. Due to these properties chitosan is able to increase the penetration into the vaginal tissue cells, thereby making it capable for vaginal drug delivery.^65^ Diego et al developed chitosan loaded hydrogel system to treat vaginal co-infection. They prepared hydrogels containing 1% w/w chitosan in free form or as NPs. They found that these improved the mucoadhesiveness and improved the effect against the co-infection which concluded that this system is a feasible method for the treatment for the vaginal co-infection.^66^ Beatriz et al prepared clotrimazole loaded into poly (d,l-lactide-*co*-glycolide)-chitosan-NPs for treatment of vaginal infections generated by *Candida albicans*. They found that chitosan enhanced the antifungal activity and mucoadhesive properties thereby coming up as a potential treatment for vaginal applications.^67^

## Cancer therapy

Cancer is characterized by high degree of adaptability and flexibility.^68^ With developments, chemotherapy has been advanced to targeted therapy which targets the growth factor receptor instead of the toxic agents.^69^ Few are compiled in Table 3.^70-73^ Despite of all the improvements, drug resistance has developed resulting in failure of the existing methods.^74^ Boroujeni et al developed curcumin loaded folate-modified-chitosan-NPs for breast cancer therapy. They performed various tests and concluded that these NPs were potential carriers in targeting therapy for delivering curcumin to cancerous cells.^70^ Vignesh et al. prepared chitosan ascorbate NPs for the inhibition of cervical cancer. They performed various in vitro and in vivo studies and found that these NPs reduced the viability of the cervical cancer cells without affecting the viability of the normal human cells. So, this proves that these NPs are a potential system for treatment of cervical cancer.^71^ Glycol chitosan NPs based drug delivery systems have great tumor-homing efficacy. These enhance permeability and retention effect. They act by tumor targeting and improve the therapeutic efficacy.^72^ Ana Vanessa et al used epidermal growth factor receptor-targeted chitosan NPs for the delivery of cisplatin for the treatment of cisplatin resistant and sensitive lung cancer models. They performed in vitro and in vivo studies and found that this system enhanced the tumor inhibition efficacy but was surprisingly more effective is cisplatin-resistant tumors.^73^ Cánepa et al developed Interferon-alpha-2b-loaded chitosan NPs for oral drug delivery in treatment of some types of cancer and viral infections.^75^

**Table 3:** Investigations compiled based on chitosan NPs in cancer therapy

**Modifications made in Chitosan NPs**	**Drug Used**	**Inference**	**Reference**
Curcumin loaded folate modified-chitosan NPs	Curcumin	Potential carriers in targeting therapy for delivering curcumin to cancerous cells	70
Chitosan ascorbate NPs		Reduced viability of cervical cancer cells	71
Epidermal growth factor receptor-targeted chitosan NPs	Cisplatin	Enhanced the tumour inhibition efficacy but was surprisingly more effective in cisplatin-resistant tumours	72
Hydrophobically modified glycol chitosan NPs	Camptothecin	Showed marked anti-tumor effects and high tumour targeting ability.	73

## Conclusion

Chitosan NPs are effective in drug delivery and also enhance the therapeutic efficacy of the drugs. They are used in ocular drug delivery due to its in-situ gelling properties and mucoadhesive character. They are used in oral drug delivery as it opens the tight junctions of the mucosal membrane and enhances absorption. Their positive charge makes them helpful in pulmonary drug delivery. They increase the permeability of various drugs making them available for nasal delivery. They enhance the absorption of hydrophilic molecules thereby helping in mucosal drug delivery. They act as an adjuvant in vaccine delivery. These have been found to be effective in cancer therapy. Overall, these applications of chitosan NPs are due to its physiochemical properties. These include biocompatibility, biodegradability, mucoadhesive character, absorption enhancing capability and in-situ gelling property. However, a critical examination regarding the toxicity and safety issues of chitosan NPs and its manufacturing technique need to be investigated very closely.

## Ethical Issues

Not applicable.

## Conflict of interest

The authors report no conflicts of interest.
